# Smart Caching Based on Mobile Agent of Power WebGIS Platform

**DOI:** 10.1155/2013/757182

**Published:** 2013-10-28

**Authors:** Xiaohui Wang, Kehe Wu, Fei Chen

**Affiliations:** ^1^Postdoctoral Mobile Research Station of Management Science and Engineering, North China Electric Power University, Beijing 102206, China; ^2^School of Control and Computer Engineering, North China Electric Power University, Beijing 102206, China

## Abstract

Power information construction is developing towards intensive, platform, distributed direction with the expansion of power grid and improvement of information technology. In order to meet the trend, power WebGIS was designed and developed. In this paper, we first discuss the architecture and functionality of power WebGIS, and then we study caching technology in detail, which contains dynamic display cache model, caching structure based on mobile agent, and cache data model. We have designed experiments of different data capacity to contrast performance between WebGIS with the proposed caching model and traditional WebGIS. The experimental results showed that, with the same hardware environment, the response time of WebGIS with and without caching model increased as data capacity growing, while the larger the data was, the higher the performance of WebGIS with proposed caching model improved.

## 1. Introduction

As the state grid corporation “three sets of five” (intensive management in human resources; financial resources; and material resources; large-scale movements in programming, construction, operation, overhaul, and production) system construction plan is put forward, the power information construction develops toward intensive, platform, distributed shared direction. As the foundation platform, GIS should be placed in the first place during information construction. And in order to realize spatial information sharing and interoperability, the demand of integrating WebGIS in power information system is increasingly urgent [1].

Power WebGIS platform could integrate all types of equipment belonging to power enterprise, which contains power equipment, substation, transmission and substation network, power users and power load, production and management, and other core business, and form composite management system to meet the requirements (safe, reliable, high-quality, efficient, and economic operation) of power enterprise and its customers. Plan and manage the power grid by using modern technology and management means; enhance power grid equipment asset management, operation management, and regulatory capacity; improve the power supply reliability and power quality; and provide high quality, efficient, and safe service for electricity customers timely [2].

The caching technology is a common mean to solve the WebGIS space data access efficiency. In WebGIS platform, the main impact of spatial data access efficiency consists of two parts: first, database access efficiency and second, the transmission efficiency of the network [3]. Therefore, considering from the two aspects: database and network, we designed dedicated power WebGIS sharing platform cache mode land; it can greatly improve the WebGIS platform transaction processing ability and the response speed of the map. Literature 4 addresses performance issues in a systematic manner from the aspects of architecture, bottlenecks and performance factors, performance improving techniques, and performance solutions [4]. Literature 5 proposed WebGIS framework based on multiagents and made a more extensive and in-depth research on related technical problems, which overcome the defect of the traditional WebGIS in Internet environment [5]. Luo et al. introduces a multilevel componentized WebGIS system, named Geo-Union, and designed the space cache framework. It is divided into three levels: spatial database caching and network spatial caching and spatial data proxy server [6]. Literature 7 proposed a prefetching algorithm called Retrospective Adaptive prefetching, which takes the former actions of the user into consideration, to reduce the user-perceived response time and to improve user's navigation efficiency [7].

To solve spatial data access, efficiency problem existed in the process of electric power construction of WebGIS; *i* used to put forward spatial data accelerate engine in literature 8. The spatial data accelerate engine consists of spatial data model, spatial index method, and spatial index caching mechanism [8].

This paper is organized as follows: first it provides a brief description of independent research and development of electric power WebGIS platform architecture, and then it presents dynamic display cache model and agent-based dynamic display cache method, finally it verifies the proposed method; finally, conclusion and further research direction are given.

## 2. Power WebGIS Platform

Power WebGIS platform is designed based on SOA architecture and is aimed at achieving power enterprise labor and material resources intensive management and business centralized operation, using spatial data sharing and business systems integration as the starting point, to construct a service-oriented, comprehensive, and real time power system comprehensive information integration platform.

**Figure 1:**
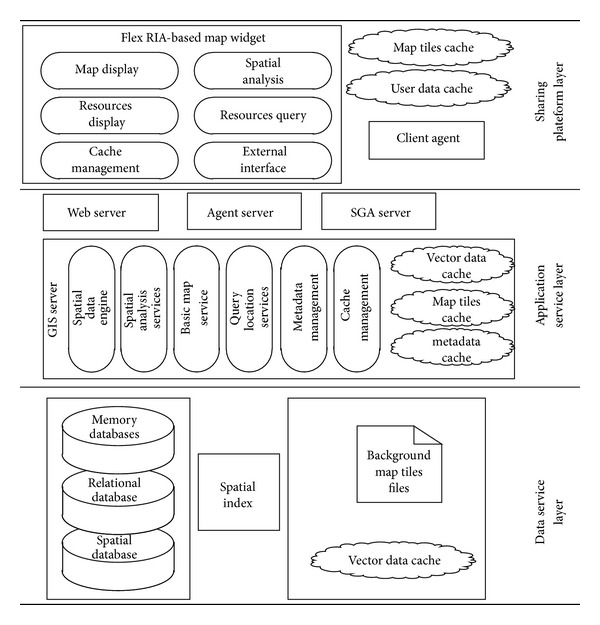
Power WebGIS architecture.

Besides the general function and the performance of WebGIS platform, Power WebGIS platform also needs to meet the requirement of electric power industry. Such as integrating electric power business data, providing power characteristic service and providing secondary development function to support related business systems integration at the same time; power WebGIS platform architecture is shown in Figure 1.

The data service layer is at the bottom of power WebGIS platform; it contains all kinds of data mentioned in the spatial data model such as spatial index for spatial data and database-side vector data cache and the background map tile server.

The application services layer is the core of the power WebGIS platform. It contains most application logic of WebGIS and various types of data cache Web server for client service agent server spatial information server (SGA server), Various types of functions are provided as services for the client calls. Unlike general WebGIS, we establish three types of caching service at the layer of the application logic, consisting metadata cache, the permissions cache, and data cache (background map cache and vector graphics data cache). The cache uses a unique design and can greatly improve the speed of response of the WebGIS. The cached data can also be shared between agent server, coordinated distributed deployment servers, and clients.

Platform sharing layer interacts directly with the user, in order to enhance the human-computer interaction capacity of WebGIS and improve client's performance. On the basis of general browser, based on Flex RIA technologies, one can realize the map control and caching mechanism. At the same time in order to reduce the network load and customer service network latency and improve WebGIS strength and fault tolerance, designed multiagent structure in Flex RIA achieves synergies in the client and speed up map response.

By using Flex technology and agent technology, power WebGIS platform consummate power business integration application system, while improve the user experience at the same time. In addition, Power WebGIS platform establishs two types of cache: cache separately for user data (vector data cache and metadata cache) and background map tile cache at display interface layer, which completes the maps collaborative function and cache data sharing between clients by moving agent. User interface control layer functions involve map display, map edit, resources display, resource query, resource location, spatial analysis, thematic map display, and so on.

## 3. Caching Model and Its Replacement Algorithm

### 3.1. Dynamic Display Caching Model

Dynamic display cache model is a Caching model set up basing on power GIS spatial data model, making database, GIS server, the client as the research object and caching sharing data in the way of memory and file. Its goal is to improve the response speed of the power WebGIS sharing platform and speed up the map rendering efficiency, rich client space operation level.

The formal description of the Dynamic Display Cache Model is as follows.

1. Cache Directory (CD): CD = {*M*, *F*, *T*}, where *M* is a collection of metadata cache, *F* is a collection of facilities feature cache, and *T* is the set of topological relations cache, the CD is dynamic display cached content, and the following will descript the specific content of each directory in detail.
2. The metadata cache set Metadata (*M*): *M* = {*B*, CS, SL, FM, RS, *V*}
  - Boundary (*B*): the scope of the map;
  - Coordinate Reference System (CS): map reference coordinate system, including all standard coordinate system defined in EPSG, and supports custom extensions;
  - Symbol Library (SL): the platform vector symbol library, for punctate facilities rendering;
  - Feature Model (FM): facility model FM = {FNO, *F*  type, *F*  style}, where FNO is facilities model number used to identify a type of facility, *F* type is the type of facility, possible values (point, line, surface, body, multipoint, multiline, multifaceted, and complex body), and *F* style is the facilities layer rendering style;
  - Relation Set (RS) is a set of possible relationships between the facilities, RS = {RNO, *R*  type, *Rf*_1_, *Rf*_2_}, where RNO is relationship identification number, *R* type is the type of relationship, *Rf*_1_ is the first facility number involved in the relationship, and *Rf*_2_ is second facility number involved in the relationship, if it is one-scale relationship, *Rf*_2_ = 0;
  - Version (*V*) is the metadata cached version.
3. Facilities feature cache set Feature (*F*): *F* = {*F*_0_, *F*_1_, *F*_2_,…, *F*_*n*_}, where *F*_*i*_ ∈ *F*∩*F*_*i*_⊙*FM*, one of All facilities category, which means instance, the *F* is an instance of FM. *F*_*i*_ = {*f*_*i*0_, *f*_*i*1_, *f*_*i*2_,…, *f*_*im*_}, *f*_*ij*_ ∈ *F*_*i*_, is a concrete class facilities.
4. Topological relations cache set Topology (*T*): *T* = {*t*_0_, *t*_1_, *t*_2_,…, *t*_*n*_}, *t*_*i*_ ∈ *T*, stand for a specific topological relations.
5. Cache Level (CL): CL = {cl⁡_1_, cl⁡_2_, cl⁡_3_}, where cl_1_ stand for level 1 cache, namely, database cache; cl_2_ stand for the second level cache, namely, the GIS server cache; cl_3_ stand for Level cache, namely, the client cache.
6. Cache Mode (CM): CM = {cm_1_ and cm_2_, cubic CM}, where cm_1_ stand for memory cache whose cache efficiency is high and is difficult to share and maintain; cm_2_ stand for file cache which is difficult to share synchronized copy; cm_3_ stand for memory file caching, has advantages of the two cache mentioned above, to maintain cache shared and cache consistency.

DDC model are defined as follows:

(1) $$\begin{array}{c} \text{DDC}=\text{CD}\times \text{CL}\times \text{CM}=\left\{M,F,R\right\}\times \text{CL}\times \text{CM}. \end{array}$$

The creation and updating of cache is closely related to the version number stored in the metadata, and low-level cache relies on high level cache, take level 3 caches for example, to demonstrate the process of cache updating

 if (CD(cl_3_) is null), then create (CD_*i*_, cl_3_, cm_1_);
   else if (*V*(cl⁡_3_) = *V*(cl⁡_2_)), then updateCache (cl⁡_3_),
   else dropCache (cl⁡_3_),
   create (CD_*i*_, cl⁡_3_, cm_1_),

where CD(cl_3_) represent the client cache directory, create (CD_*i*_, cl_3_, cm_1_) is to create the client cache directory, cache mode uses the memory cache, *V*(cl_3_) stand for the version number of the client cache, updateCache (cl_3_) is to update the client cache, and dropCache (cl_3_) is to empty the client cache.

Cache model creation and maintenance should ensure the integrity and consistency of cache. In order to realize the cache efficiency at the same time, some rules need to be followed.

*Rule  1*. CD must guarantee the integrity, convenient for local client to render graphics, and support simple topology analysis and spatial query.

*Rule  2*. Cache level CL can support arbitrary combination, which contains eight kinds of state, but often maintain the whole cache state so as to ensure high efficiency of the system.

*Rule  3*. CM only has single state, can only be one of the following states: memory cache, file cache, or memory file cache. Usually adhere to the following pattern: first carries on the memory cache, when the amount of cache increase to set limit, based on sliding window replacement strategy, transfer a part of the memory cache to memory file cache. When the client is closed, transferred into the file cache.

### 3.2. Caching Replacement Algorithm

Caching replacement algorithm is established with space limitation and time limitation as theoretical basis. Space limitation behaved as if the most remote distance eliminated first, and time limitation behaved as if the longest time unvisited eliminated first. Comparing with the traditional FIFO, LRU, and LFU, we chose 2Q (two queues algorithm) to improve the efficiency of caching replacement.

2Q algorithm does not eliminate the page least visited from main cache but achieves through swapping with the page most visited. Similar with LRU/2 algorithm, 2Q distinguishes the pages with the time visited the second time, that is to say, 2Q puts the page first visited into a special cache called *A*_1_ queue, which is a FIFO queue, and move it to *A*_*m*_ queue if the page is visited again in the life cycle, which is LRU, likewise, swap it out if it is not visited in the life cycle of *A*_1_ queue.

In 2Q algorithm, *A*_1_ queue exists as a filter, the data could swap to *A*_*m*_ queue when it is visited again after entering the *A*_1 out_ queue. Assuming miss rate is *m* and 1 out queue space is *f*, so the data could be swaped to *A*_*m*_ queue if it is visited less than *f*/*m* after it entered the *A*_1 out_ queue, we called the probability *p*_accept_. Assuming the rate of object *i* visited is *p*_*i*_, then the rate that it was swapped to *A*_*m*_ from *A*_1 out_ after the visited *k* time is *p*_*i*_(1 − *p*_*i*_)^*k*−1^, and *p*_accept_ could be achieved as

(2) $$\begin{array}{c} p_{\text{accept}}=\overset{f/m}{\underset{k=1}{\sum }}{p_{i}\left(1-p_{i}\right)}^{k-1}=1-{\left(1-p_{i}\right)}^{f/m}. \end{array}$$

*p*_accept_ is close to 1 as *p*_*i*_ increased, while close to 0 as *p*_*i*_ decreased, so *A*_1 out_ plays a role of filtering the page least visited. We could define the cutoff hotness of *A*_1 out_ filter as *p*_cutoff_, in the case of *p*_accept_ = 1/2, the derivative of *p*_cutoff_ is very large, so the object with the visiting rate *p*_cutoff_ could be swapped into *A*_*m*_ after it was visited the third time not the second time; the visiting rate could be achieved according to (3). If the *f* and *m* are given, *p*_cutoff_ could be expanded as (4) with Taylor series expansion:

(3) $$\begin{array}{c} {1-\left(1-p_{i}\right)}^{f/m}=\frac{1}{2}, \end{array}$$

(4) $$\begin{array}{c} p_{\text{cutoff}}=1-{\left(\frac{1}{2}\right)}^{f/m}\approx \frac{m\ln2}{f}. \end{array}$$

Formula (4) shows that *A*_1 out_ filter could self-adjusted, *p*_accept_ will be relatively large when miss rate is large, so the process will swap the object most visited, while the object usual visited could be swapped in when miss rate is low. Assuming *p*_*i*_ and *m* are given, *f*_crit_ could be achieved in the condition of *p*_accept_, as

(5) $$\begin{array}{c} f_{\text{crit}}=\frac{-m\ln2}{\ln\left(1-p_{i}\right)}\approx \frac{m\ln2}{p_{i}}. \end{array}$$

The established condition of (5) is ln⁡⁡(1 − *p*_*i*_) ≈ −*p*_*i*_, and *f* could be achieved when visited rate limit of *A*_*m*_ queue is given. Assign the value of *f* = *f*_approx_, average visited object could be swapped into *A*_*m*_ queue. If miss rate is *m* and size of *A*_*m*_ is *B*, the average visited rate of object in the buffer is (1 − *m*)/*B*, and formula (6) could be achieved when replacing *f*_crit_ with *f*:

(6) $$\begin{array}{c} f_{\text{approx}}=\frac{\ln\left(2\right)mB}{1-m}. \end{array}$$

The practical significance of (6) is limited as the miss rate need to be estimated, but it illustrates that the ratio of *A*_1 out_ size and buffer size is *B*. Simulation results showed that the ratio is 1/2 when the value of m is from 10% to 90%, and *A*_1 out_ size is smaller if the m is smaller. Assuming *D* as the whole data set and *B* = *rD*, *A*_*m*_ is part of *D*, shown as (7), then the average visited rate of the object least visited is *m*/(1 − *r*)*D*. Assuming *X* = *m*/*D*, the average visited rate of the object least visited is (1/(1 − *r*))*X*, which is

(7) $$\begin{array}{c} p_{\text{cutoff}}=\frac{2m\ln\left(2\right)}{rD}, \end{array}$$

(8) $$\begin{array}{c} p_{\text{cutoff}}=\frac{2\ln\left(2\right)}{r}X. \end{array}$$

Because *r* is very small, so (2ln⁡⁡(2)/*r*)*X* ≫ 1/(1 − *r*). The object least visited could be filtered as long as *r* is small enough with the condition *f* = *B*/2; moreover, *f*_approx_/*B* = 1/2 could result *m* = 1/(1 + 2ln⁡⁡(2)) ≈ 0.419. As long as *m* is not large enough, the fluctuation of *f*_approx_ will not be large, so the algorithm is not sensitive to the setting of *f*, and *f* = *B*/2 is always right.

## 4. Dynamic Display Cache Based on the Agent

Power GIS model has the following characteristics: complex, large amount of data, high real-time requirements, history play back, and real-time tracking operations. So multilevel cache on spatial data can effectively reduce the server pressure and network load, achieve efficient and real-time access to spatial data. It is also critical to improve the efficiency of the system and reduce the map response time [9].

**Figure 2:**
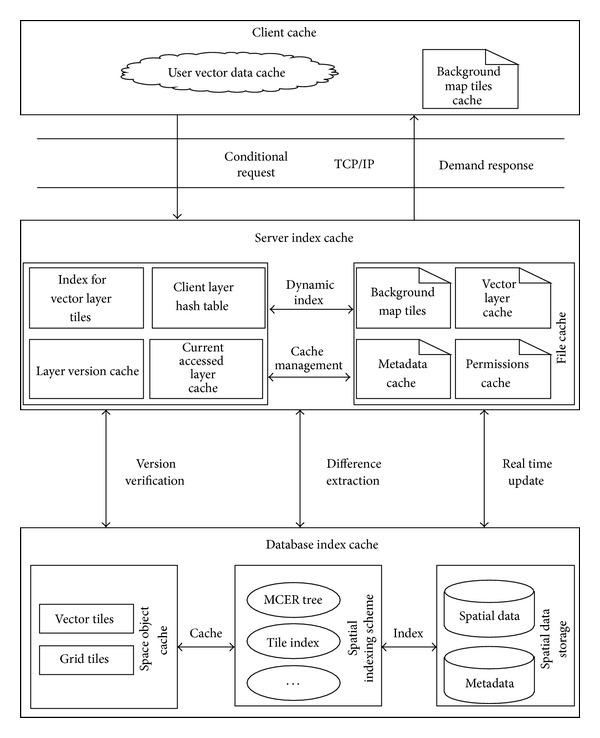
Power WebGIS dynamic display cache.

In order to quickly and effectively deal with huge amounts of spatial data stored in the space database, we implement three-level cache in Power WebGIS platform including the database-side, server-side, and client. The platform dynamic cache model is shown in Figure 2. Create a spatial index and tile cache on database-side and improve query efficiency by querying attribute data instead of the space data. Through the management, the vector cache server, and the map tile server, server-side caches dynamic map layers vector data, user data and layer version data using high-speed dynamic display cache (DDC). Server-side also caches background map in the form of tile data cache. Through a distributed deployment strategy, it reduces network traffic, speeds up the access speed, greatly improves the concurrent user response ability, and realizes high stability of the cache. Through the intelligent agent technology, the client communicates with server-side cache by adopting asynchronous transfer technology, set different cache according to the access level and access frequency, realize sensitive interaction and rich operation experience, improve the efficiency of response.

Server-side dynamic display high-speed cache, and background map tiles cache can satisfy power facilities data real-time change and meet vector graphics rendering requirements. The system can quickly generate maps without query background database so it still has high efficiency while managing huge amounts of data at the same time. The flexible cache can be placed anywhere in the network, considering the system performance, storage space, and network traffic. To meet the requirement of real-time updates, we use DELTA mechanism, update dynamic information through incremental form. Through the combination of DDC and DELTA, it can better support huge amounts of data, accelerate the access speed and ensure data correctness.

The client cache is based on the following fact: in a period of time, user's inspection of the map and the retrieval of power facilities are concentrated in certain layer. According to the access frequency of the layer, the data is cached in the different levels of cache, when a user retrievals data start from the fastest cache. If does not exist, then retrievals in the next cache. Client local caching content includes all or part of the DDC, the background map tiles visited, all kinds of versions of space facilities data model, user permissions metadata, and others. When facilities updated, it realizes real-time requirements by the server-side DELTA mechanism.

By applying multiagent technology, the power WebGIS platform client provides basic maps show function, browse function, space analysis function, resources query localization function, local map cache data, and user data management function. In addition, each client registers a client agent, as the link between the clients completes the function of map synergy and cached data sharing between the clients by mobile agent.

**Figure 3:**
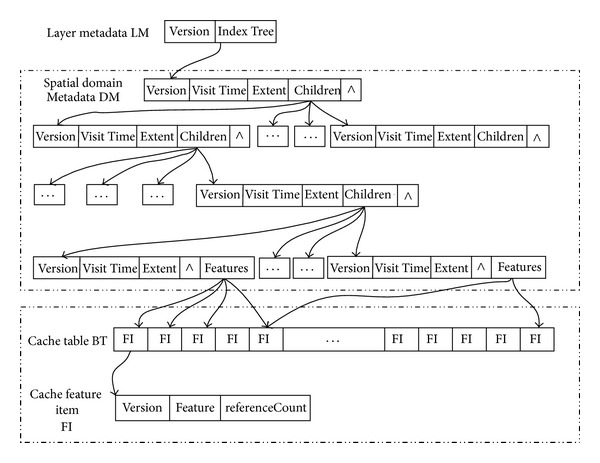
Cache data model.

Dynamic cache mechanism based on intelligent agent creates cache spaces on the server-side and the client-side, respectively. Server-side cache is maintained by multiple applications terminal jointing, while the client cache is maintained by each application terminal separately. The caching model adopts version-based caching strategy according to the layer version, the version of the spatial domain feature, and the version of cached data sheets. Due to the quad-split, relationship exists between the spatial domains trellises coded, so when retrieving in the index tree, we can skip certain levels of spatial domain node and query directly to a leaf node. Cache model is shown in Figure 3.

LM represents the layer data block metadata; version is the number of the version for the cache layer elements sets; index Tree is the quad index tree for finding spatial elements, the type of each node of the index tree is domain metadata. A one-to-one relationship exists between each node and a quad-split map (spatial domain). The maximum depth of the search tree equals to the zoom level supported by the platform.

DM represents spatial index tree node, version is the version number of cache space domain element sets; visit time represents the last access time of the spatial domain used in the LRU algorithm; extent represents the scope of the space domain used in priority replacement algorithm based on farthest distance; children represents the pointer pointing to the subspace domain; and features is used to save entry address of feature set in the region.

The feature collections are saved in the cache data sheet Buffer table.

BT represents data sheet, featureColl is cached data collection, the features cached in the data table can be shared by all spatial domain.

FI represents cached feature items model, feature represents the cached elements; version represents the version of the cached feature items; and referenceCount stand for the times a feature is referenced. When the counter is 0, the occupied storage space can be recycled.

Based intelligent agent dynamic caching mechanism, where the power WebGIS platform is able to meet the efficient data access and real-time requirements, significantly improve the hit rate of the data and effectively accelerate the speed of data access and graphics rendering efficiency.

## 5. Experimental Results

According to the dynamic cache model proposed in this paper, we design the test for vector data cache. That is to say, the elements in the vector layer cache are to be tested. Database cache refers to the unique index created on the feature id field of feature table. Create cluster index so as to index features by spatial domain (tiles) id field. Server-side and client cached data means to establish the mapping between layer and space domain set in layer; the mapping between spatial domain and features set entry address buffered in spatial domain to improve features search efficiency.

*Test Case  1*. Performance comparison with same concurrent users and different data size.

- Test object: low-voltage power lines of a provincial power company.
- Hardware environment: shown in Table 1.
- Test data size: 80000 features on sever-side and 20000 features on client-side.
- Test mode: 10 client access WebGIS server concurrently.
- Test goal: comparing the vector data loading time when using dynamic display cache model proposed in this paper with the vector data loading time without caching pattern.

Table 2 shows platform performance comparison in our test. As can be seen from the comparison results, dynamic display caching can shorten the system response time by 2–5 times. Results show that the proposed dynamic display cache technology can significantly improve the response speed of the map and enhance the user experience. 

*Test Case  2*. Response time comparison with same data size and different concurrent users.

- Test object and hardware environment was the same as test case 1.
- Test data size: 4000 features.
- Test mode: 10,20,30,…, 100 concurrent user.
- Test goal: comparing the vector data loading time with dynamic display cache model proposed in this paper with the vector data loading time without caching pattern in different concurrent users.

**Figure 4:**
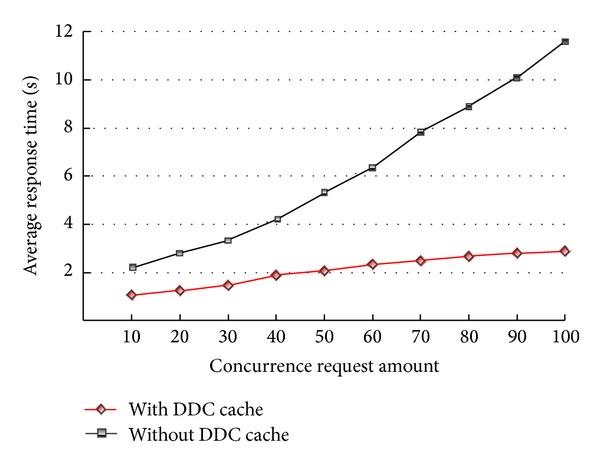
Average response time using different number of concurrency request.

Figure 4 showed the experimental results, from which we could conclude the following.

1. The response time of the two ways was low with the smaller concurrent users, but the response time of noncaching mode was 2 times that of caching mode.
2. The response time of the two ways both increased as the concurrent users added, but the response time of noncaching mode increased faster, and the response time of noncaching mode was 4.2 times than that of caching mode when the concurrent users reached 100.
3. The response time could be lower obviously when the dynamic display caching model was used, while the advantage with caching mode was greater and greater as the concurrent users. Dynamic display caching could shorten the system response time by 2–4.2 times.

## 6. Conclusion

Aiming at the key problem affecting the power performance of WebGIS-caching mechanism, this paper presented a dynamic display cache model, which makes a research on dynamic display cache technology based on intelligent agent, designs the power WebGIS dynamic cache structure, and cached data model. The application of the intelligent caching technology can greatly improve the WebGIS graphics loading speed and the response efficiency when dealing huge amounts of data, improve user concurrent traffic, and balance the network load. But research is still insufficient; the future research direction is described below.

1. Smart cache can improve the loading speed of vector graphics and meet the needs of two-dimensional GIS, but for Digital Elevation Model (DEM), aerial data, there are no efficient load acceleration solutions.
2. Mobile agent can improve the client cache sharing and improve the response speed of the map, but further study on cache data security and the mobile agent secure communications is needed.

## Figures and Tables

**Table 1:** Hardware environment.

Item	Server	Client
OS	Windows server 2003	Windows 7
CPU	Intel(R) i3	Intel(R) i3
Memory size	4 GB	2 GB
CPU Clock Speed	2.4 GHz	2.4 GHz

**Table 2:** Performance comparison.

Vector data number	Nonindexed cache mode load time (ms)	Indexed cache mode loading time (ms)	Load faster ratio
1,541	942	320	2.94
2,075	1,245	805	1.54
4,111	2,421	1,167	2.10
9,215	5,381	1,675	3.21
17,598	9,472	2,950	3.21
40,393	21,364	5,089	4.19
78,980	45,898	9,374	4.89
141,481	98,507	21,569	4.58
